# Supplementary material for: Microbial fertilizers modulate tobacco growth and development through reshaping soil microbiome and metabolome
Source: Microbiol Spectr. 2025 May 22;13(7):e02605-24. doi: 10.1128/spectrum.02605-24 (PMC12211045; doi:10.1128/spectrum.02605-24)
Supplement: Supplemental material — Fig. S1 to S5. [file spectrum.02605-24-s0001.docx]

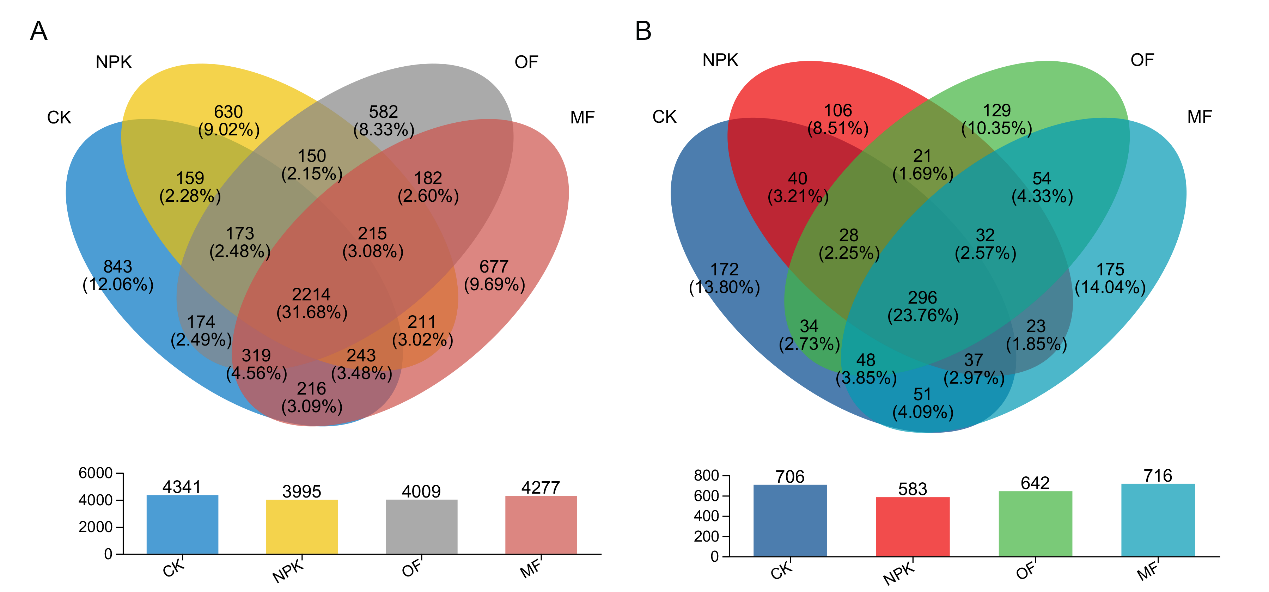


Figure S1. Venn diagrams and bar plots illustrating the shared and unique OTUs among different fertilization treatments for bacterial (A) and fungal (B) communities.


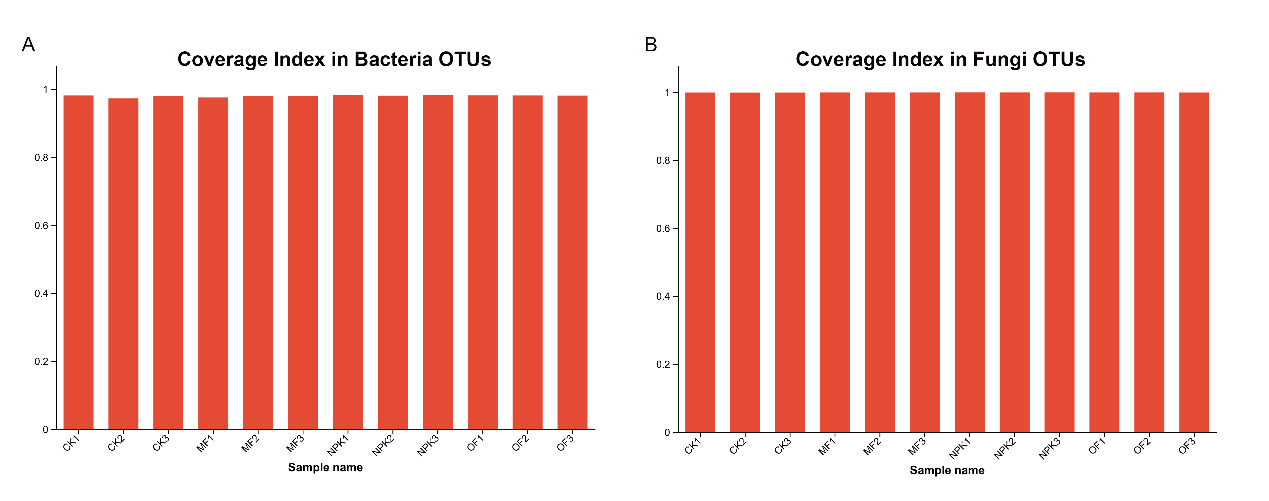


Figure S2. Coverage indices for bacterial (A) and fungal (B) OTUs.Values closer to 1 indicate that the sequencing depth was sufficient to capture the majority of microbial taxa in the community.


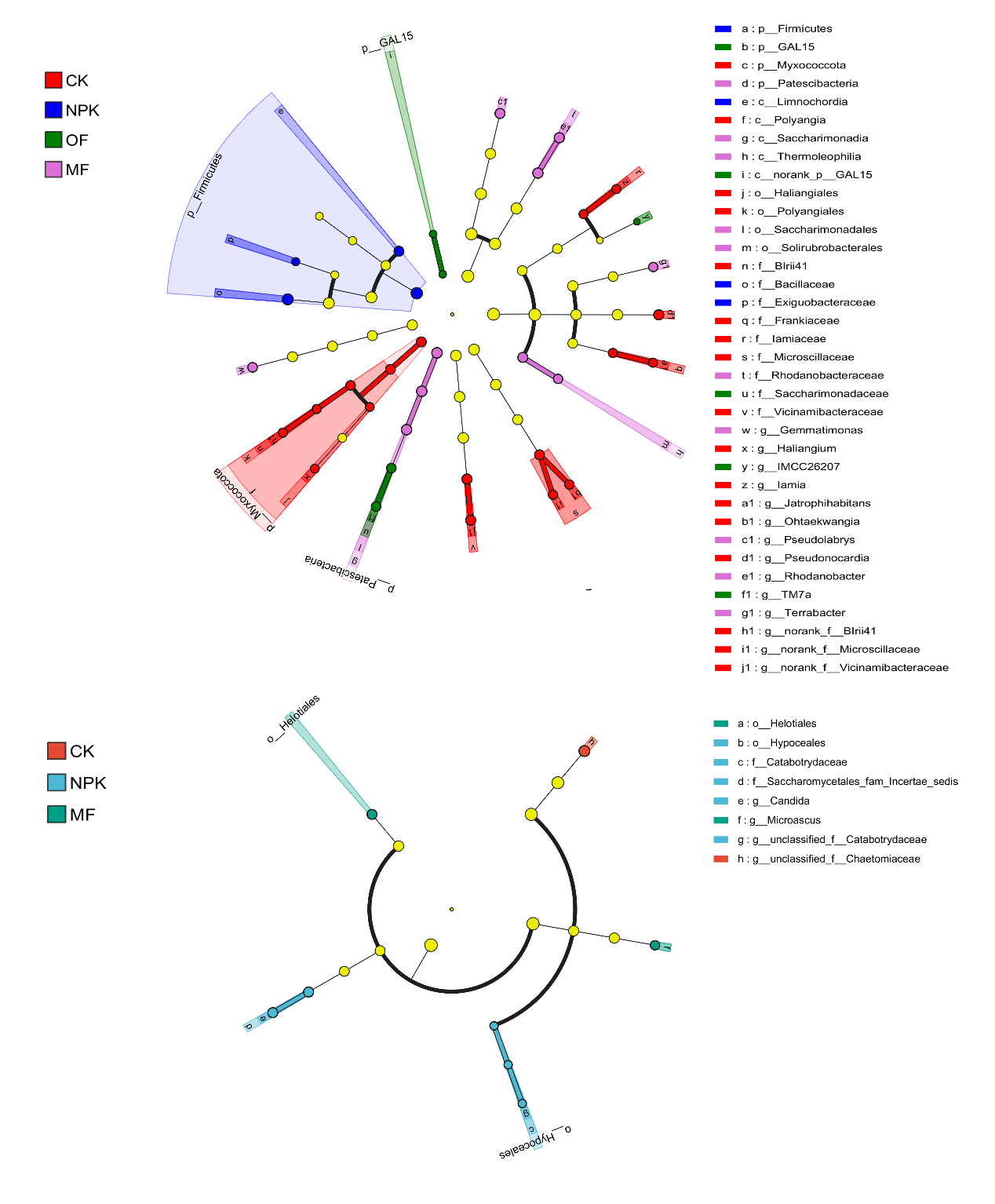


Figure S3. LEfSe Multilevel Species Difference Discriminant Analysis (multilevel: phylum, class, order, family, genus, species) Tests of difference were performed at multiple levels to analyze differences at multiple levels: (A) bacteria, (B) fungi (LDA > 3).


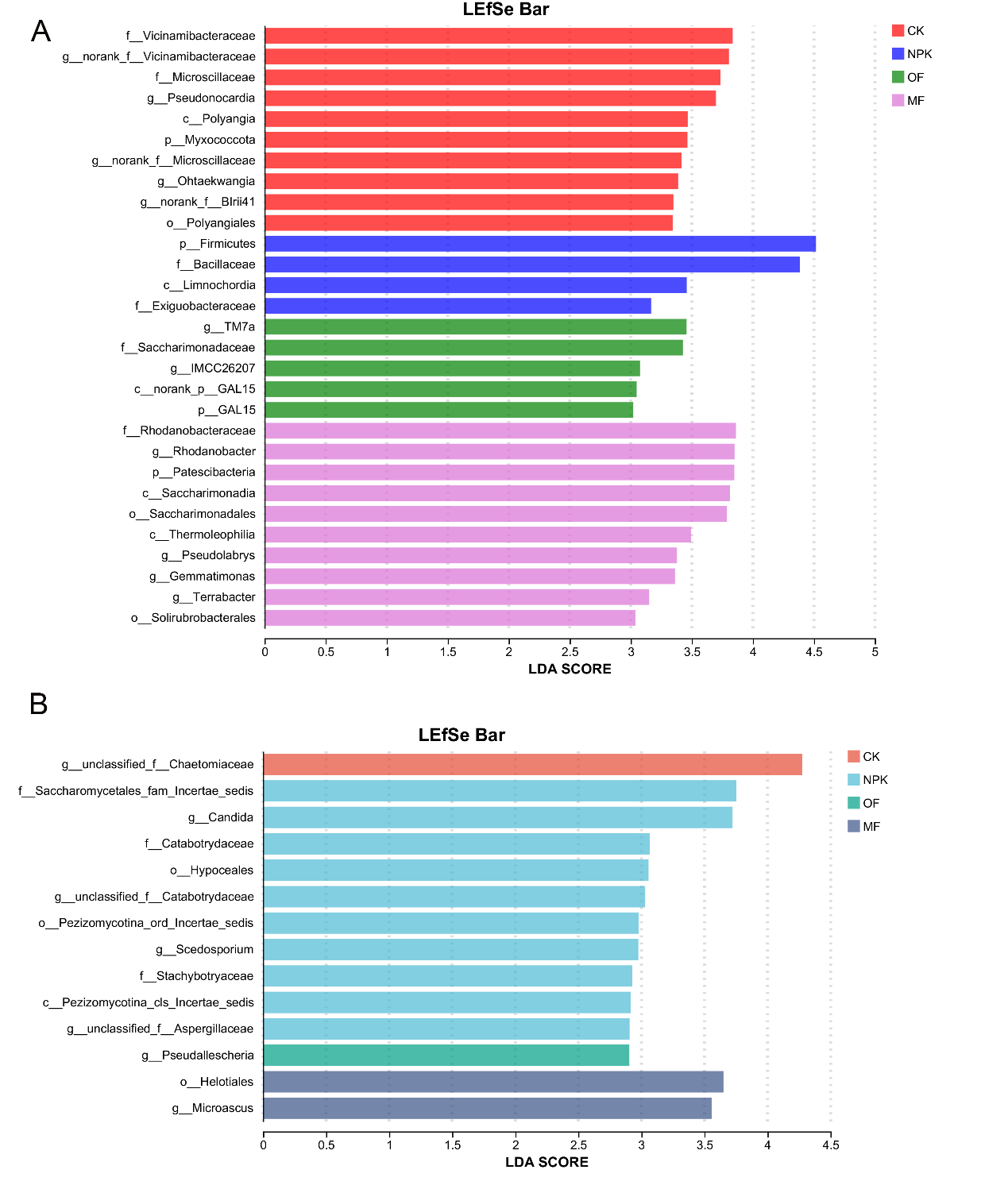


Figure S4. LDA discriminant bar chart statistics of microbial taxa with significant effects in multiple groups, LDA scores obtained by LDA analysis (linear regression analysis), the larger the LDA scores, the greater the impact of species abundance on the differential effects: (A) bacteria, (B) fungi.


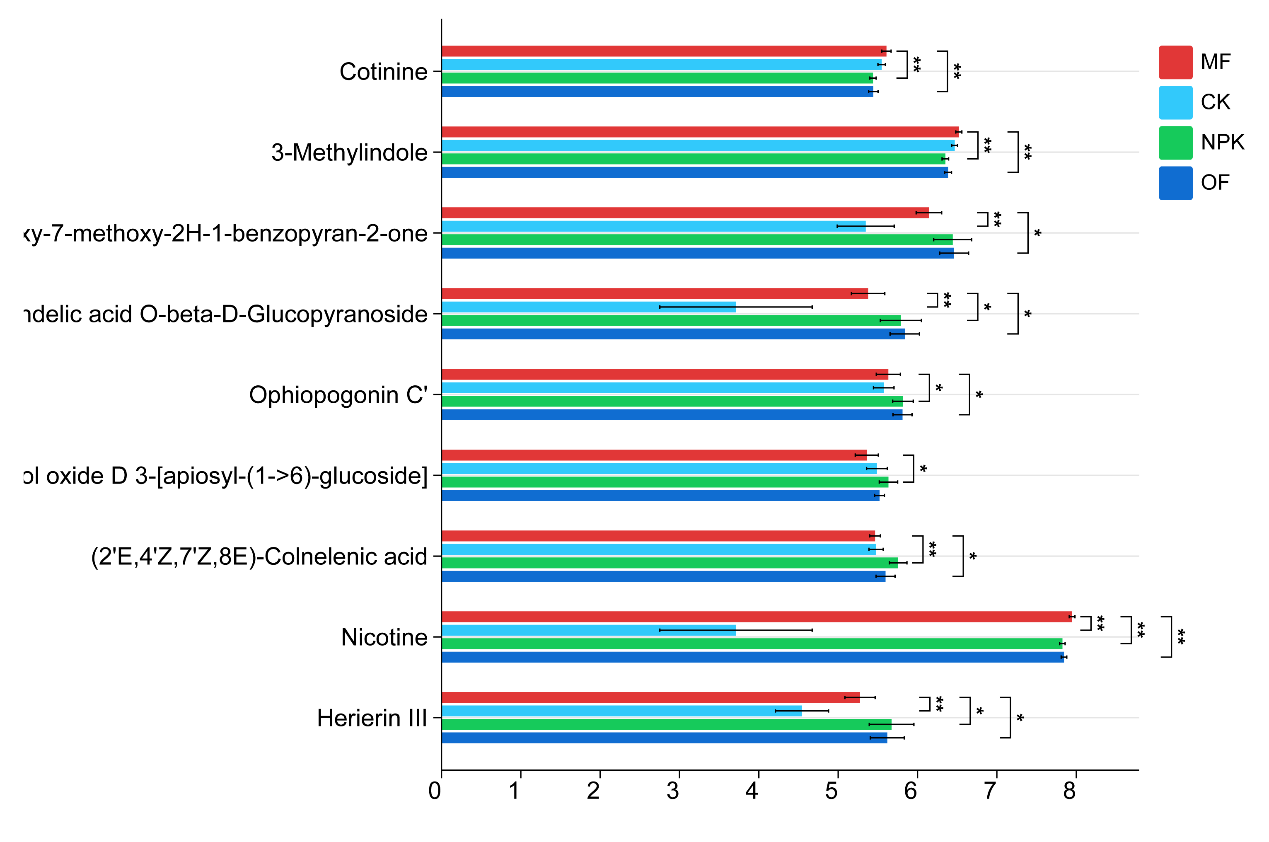


Figure S5. Bar graph comparison of 9 significantly different metabolites in CK, NPK,OF,MF
